# Preparation and *in vivo* evaluation of an intravenous emulsion loaded with an aprepitant-phospholipid complex

**DOI:** 10.1080/10717544.2023.2183834

**Published:** 2023-02-27

**Authors:** Yan Li, Hong Yin, Chensi Wu, Jia He, Chunyan Wang, Bo Ren, Heping Wang, Dandan Geng, Yirong Zhang, Ligang Zhao

**Affiliations:** a Department of Pharmacy, Tangshan Maternal and Child Health Hospital, Tangshan, China; b School of Pharmacy, North China University of Science and Technology, Tangshan, China; c Department of Pharmacy, Hohhot Hospital of Traditional Chinese Medicine and Mongolian Medicine, Hohhot, China; d Tangshan Key Lab of Novel Preparations and Drug Release Technology, Tangshan, China

**Keywords:** Aprepitant, lipid injectable emulsion, response surface methodology, pharmacokinetics, tissue distribution

## Abstract

In present, there was no detailed report on the formulation optimization and quality evaluation of aprepitant (APT) injectable lipid emulsion (APT-IE). The aim of the present investigation was to prepare and evaluate its properties of APT-IE loaded with an APT phospholipid complex (APT-PC) *in vitro* and *in vivo*. APT-PC was obtained by solvent evaporation with APT and phospholipids, then analyzed by *X*-ray diffraction, Fourier transform infrared spectroscopy and differential scanning calorimetry. Lipid emulsions are a new formulation that can reduce side effects and improve drug loading.

APT-IE prepared by High-pressure homogenization and optimized by response surface methodology (RSM). The proportion of sodium oleate, poloxamer 188 and soybean oil were selected as variables for the optimization. The optimal formulation of ATP-IE had the following characteristics: particle size, 82.83 ± 1.89 nm; polydispersity index, 0.243 ± 0.008; zeta potential, −59.0 ± 2.54 mV; encapsulation efficiency, 98.84%±1.43%; drug loading, 7.08 ± 0.16 mg/mL; and osmotic pressure, 301 ± 2.15 mOsmol/kg. Transmission electron microscopy images indicated that the particle diameter of APT-IE was approximately 100 nm, with a morphology of spheroidal or spherical. APT-IE exhibited sufficient stability after storage at 4 ± 2 °C for more than 6 months. The results of the pharmacokinetic study demonstrated that APT-IE had the advantages of better safety, higher bioavailability, and obvious liver targeting than APT solution (APT-SL). The area under the curve (AUC) of APT-IE was 3-fold enhanced compared with APT-SL. The targeted enhancement multiple of APT-IE to liver tissue was greater than that of APT-SL. These results suggested that APT-IE has broad clinical application and industrial production potential.

## 1. Introduction

From the perspective of the patients, the most unpleasant side effects of cancer chemotherapy are vomiting and nausea. Poor control of these side effects can lead to a decline in quality of life (Ballatori & Roila, 2003). At present, chemotherapy-induced nausea and vomiting (CINV) is mainly prevented by recombination of 5-hydroxytryptamine receptor antagonist (5-HT_3_RA) with dexamethasone, which still fruitlessly delays CINV (Zhang et al., 2020). Aprepitant (APT) is the only neurokinin-1 receptor antagonist (NK-1, RA) authorized for the prevention of moderately and highly emetogenic chemotherapy-induced CINV (Aapro et al., 2015; Ottoboni et al., 2018). APT is usually combined with other antiemetic drugs in order to prevent acute or delayed CINV (Zhang et al., 2020). Moreover, it can suppress nausea and polyneuropathy semaphore by preventing substance *P* from interacting with the NK-1 receptor (Tsukiyama et al., 2018). However, poor water-solubility, first-pass metabolism and poor intestinal mucosal permeability may cause low oral bioavailability of APT restrict its application (Hörter & Dressman, 2001; Sato et al., 2014). A few years later, forsaprepitant which was a prodrug of APT, was discovered and authorized for intravenous administration to prevent CINV in 2008. Forsaprepitant is more soluble than APT in water but still uses a powerful surfactant (Tween 80), which gives rise to serious allergic reactions and adverse events (Wang et al., 2020).

Lipid emulsions with soybean oil as the oil phase and lecithin as a surfactant have been used clinically in the USA for more than 50 years (Zhang et al., 2018). Lipid emulsions have been used as carriers for the delivery of insoluble drugs such as anesthetics (etomidate, propofol) and cardiovascular medicine (nimodipine microemulsion), in the last year (Driscoll, 2006). Moreover, Lipid emulsion as a fresh and progressive drug delivery approach has the following advantages: (1) high bioavailability, fast onset and small individual differences; (2) high solubility and stability of water-insoluble drugs, and long-lasting pharmacological effects (Li et al., 2013); and (3) lessening the venous toxicity of lipophilic drugs (Carpentier & Dupont, 2000).

Then, CINVANTI®, a novel formulation of APT emulsion without Tween 80 or other synthetic surfactants, was endorsed to prevent acute or CINV (Navari & Mosier, 2019) However, few reports about APT emulsion formulations and preparation methods are available. Recently, an APT emulsion containing cholesterol hemisuccinate (CHS) was prepared through the membrane dispersion homogenization method and sterilized by high-temperature steam (Zhang et al., 2020). However, CHS can lead to nonuniformity of lipid properties when preparing liposomes in nanosystems (Augustyn et al., 2019). The pharmaceutical safety of CHS and succinic acid should also be further investigated in clinical applications. No systematic research or detailed report on the formulation and pharmacokinetics of APT injectable lipid emulsion (APT-IE) (dose: 7.2 mg/mL) has been reported so far.

The present research is the first time to prepare aprepitant intravenous lipid emulsion with the similar physical and chemical properties as the marketed preparation and to evaluate its safety *in vitro*, pharmacokinetics and tissue distribution *in vivo*, which provides a possibility for the preparation of other drugs with similar properties and new dosage forms of APT. As APT with low permeability and poor solubility in intestine, the introduction of APT into intravenous submicron emulsions was an effective route for drug administration; however, the solubility of APT in the oil phase could not meet the requirement of drug loading of the target formulation.

In this research, phospholipid complex technology was employed to improve the solubility of APT in the oil phase, aimed to obtain the optimal formulation of APT-IE by CCD. Then evaluate its *in vivo* pharmacokinetic and tissue distribution in rats, with ATP-SL used as a reference.

## 2. Materials and methods

### 2.1. Materials and animals

#### 2.1.1. Materials

Aprepitant (APT, purity ≥ 98.5%) was purchased from Zhongshan Yiantai Pharma Co. Ltd. (Guangzhou, China). Egg yolk lecithin and sodium oleate were purchased from Shanghai A.V.T. Pharma Co. Ltd. (Shanghai, China). Poloxamer 188 (F68), sucrose and medium-chain triglyceride (MCT) were purchased from Xi’an Tianzheng Medicinal Materials Co. Ltd. (Zhejiang, China). Ethanol was purchased from Shandong Long Yu Quan Pharmaceutical Excipients Co. Ltd. (Shandong, China). All chemicals and reagents were of analytical or HPLC grade.

#### 2.1.2. Ethical approval statement

All Sprague-Dawley rats were obtained from the Experimental Animal of North China University of Science and Technology (Tangshan, China). The animal license number was 2019-0006. Each animal was breaded in a clean environment with enough water and food. The experimental protocols for laboratory animals were evaluated and approved by the University Ethics Committee and complied with the Guide for the Care and Use of Laboratory Animals. All experiments were conducted in accordance with the principles of the European Community Guidelines for the Use of Laboratory Animals.

### 2.2. Physicochemical properties of APT

#### 2.2.1. APT solubility

Excessive amounts of APT were added to 10 mL of soybean oil (SO), corn oil, MCT, SO-MCT mixture, and phosphate buffered solution (PBS) of pH 2–8. The samples were incubated for 48 h at 37 °C in a water bath vibrator after 15 minutes of ultrasonication. The mixture was filtered through a 0.45 μm membrane, with the initial filtrate discarded. Then the concentration of APT was determined by HPLC after diluted appropriately with methanol.

#### 2.2.2. Partition coefficient

The classical shake flask method was used for the determination of oil-water partition coefficient of APT. The Solution that *n*-Octanol and distilled water fully mixed was left to stand for 1 h to ensure separated completely (Peng et al., 2010). Excess APT was added to 2 mL of saturated n-octanol, then 14 mL of water was added and the mixed solution was shaken for 24–36 h. The *n*-octanol phase was diluted to 1:20 (*v/v*) with methanol, with the water phase directly postmembrane. The APT concentration in both phases were analyzed by HPLC and the partition coefficient was calculated as follows:

(1) $$\;\mathrm{Log}\;P=\frac{c_{0}}{c_{w}}$$

*Co-*the concentrations of APT in *n*-octanol, *Cw-* the concentrations of APT in water.

### 2.3. APT phospholipid complex (APT-PC) investigation

#### 2.3.1. Preparation of APT-PC with different ratios

The APT and EL were dissolved in 2 mL of absolute ethanol with rotary evaporation at 70 °C to obtain dried APT-PC. Moreover, the APT, EL or the dried APT-PC were weighed before and after preparation to ensure anhydrous ethanol completely evaporated. The ratios of APT to EL were settled at ratios of 1:1, 1:2, 1:6, 1:10, 1:15 and 1:17.5 (w/w), and the drug loaded of the phospholipid complex was used to evaluate the optional rate of APT-PC with different proportions. Moreover, the drug loaded in the phospholipid complex was also expressed as the recombination rate (Rer). The calculation of the Rer is as follows:

(2) $$\text{Rer}=\frac{w_{1}}{w_{0}}$$

*w_1_* -The concentrations of APT in APT-PC determined by HPLC.*w*_0_ -the amount of APT (0.72 g).

#### 2.3.2. Characterization of APT-PC

The properties of APT-PC were characterized by the following three ways. First, samples including free drug (APT), free phospholipid (EL), APT and EL physical mixture (APT-EL-PM) and different mass ratios of APT-PC (1:1, 1:2, 1:6, 1:10, 1:15, and 1:17.5) were prepared. For X-ray diffraction (*XRD*) analysis, samples were acquired at room temperature on a D/max-rA (Rigaku Ultima III, Japan). *XRD* setup parameters were 40 kV and 30 ma (1.2 KW) Cu K_ɑ_ source. Samples were measured by Fourier transform infrared (*FTIR)* analysis. The spectral range is set to 4000–400 cm^−1^. Using differential scanning calorimeter (DSC) analysis, samples were examined using a DSC instrument to test, verify their thermal characteristics and obtain DSC curves. Samples were put into aluminum pans and heated from 25 °C to 300 °C under a nitrogen atmosphere at a rate of rise of 10 °C/min.

### 2.4. Formulation optimization of APT-IE

#### 2.4.1. Univariate experiments

The oil phase containing corn oil, castor oil, SO, MCT and SO-MCT mixture were selected with the particle size and viscosity of APT-IE as the index. The egg yolk lecithin amount was screened basing the entrapment efficiency of the APT-IE. We ensured that the optimizing formulation was prepared to meet the drug loading and encapsulation efficiency requirements.

#### 2.4.2. CCD-RSM

The APT-IE formulation was designed and optimized using the central composite design response surface methodology (CCD-RSM). According to the results of single-factor experiments, three independent variables were designed of the proportion of sodium oleate (X_1_, 0.05–0.7%), poloxamer 188 (X_2_, 0–1.2%), and oil (X_3_, 8–18%) used. The aim of this study was to reduce the size of APT-IE. Due to smaller emulsion droplets undergo reduced Brownian movement, the emulsion is more stable. In addition, the drug absorption can be enhanced with smaller particle sizes and thus realized the greater improvements of bioavailability. The 20 runs designed by CCD were presented in Table 1.

**Table 1.** Central composite design for the study of independent variables with experimental results.

Run No.	Actual values of independent variables			Values of response variable(*Y*)
	*X_1_*	*X_2_*	*X_3_*	
1	0.38	0	13.0	85.59
2	0.38	0.60	13.0	92.59
3	0.38	0.60	8.00	93.51
4	0.57	0.96	10.03	74.52
5	0.38	0.60	13.00	91.95
6	0.38	0.60	13.00	92.57
7	0.38	1.20	13.00	59.52
8	0.57	0.24	15.97	73.62
9	0.57	0.24	10.03	94.25
10	0.70	0.60	13.00	90.62
11	0.18	0.24	10.03	94.68
12	0.38	0.60	13.00	90.95
13	0.38	0.60	13.00	89.12
14	0.18	0.96	10.03	63.52
15	0.18	0.24	15.97	71.21
16	0.38	0.60	13.00	92.13
17	0.38	0.60	18.00	75.21
18	0.05	0.60	13.00	77.62
19	0.57	0.96	15.97	81.32
20	0.18	0.96	15.97	61.52

### 2.5. Preparation of APT-IE

The APT-IE was prepared by the method of high-pressure homogenization. Firstly, APT-PC was dissolved in the oil phase. Meanwhile, sodium oleate, glycerol and poloxamer 188 were dissolved in water for injection as the aqueous phase. Then sonicated at 60 °C for 10 min to make the solution became homogenous. Secondly, the oil phase and aqueous phase were carefully mixed, with magnetic string for 10 min at 600 rpm. Subsequently, the coarse emulsion was prepared by high-speed shearing for 10 min. Finally, APT-IE was prepared using M-110EH high-pressure homogenizer (Microfluidics Corporation, MA, USA). The final emulsion was filled into vials under nitrogen protection and sterilized by 0.21 μm Nalgene® nylon filter.

### 2.6. Characterization of APT-IE

#### 2.6.1. Physicochemical properties

The particle size, PDI and zeta potential of APT-IE were determined by the principle of dynamic light scattering (Malvern, Nano Series ZSE, Malvern Instruments Co. Ltd, ZS90, UK) after APT-IE was diluted with ultrapure water. The pH of APT-IE was obtained by pH meter (Mettler-Toledo International Co. Ltd, S210-K, USA), and the osmotic pressure was determined with a Model 3250 osmometer (3250s, Advanced Co. Ltd, USA).

The drug content of APT was determined by HPLC with an Agilent C_18_ column at 30 °C. The mobile phase consisted of acetonitrile-water (50:50, v/v) at a flow rate of 1.0 mL/min, then the wavelength was set at 210 nm. The DL was calculated by Equation (3). Took 1 mL of APT-IE into 100 mL volumetric flask, with methanol added to break the emulsion. Then the drug concentration was measured by HPLC.

(3) DL=C×100

*C*: concentration of APT, the units of *C* and *DL* are both mg/mL.

The encapsulation efficiency (*EE*)% of APT-IE was measured by the Sephadex column filtration method. According to the APT-IE elution curve, partial eluent of the emulsion was collected and diluted with methanol. The following equation was used to calculate the *EE*%:

(4) $$EE=\frac{Wb}{Wa}\times 100\%.$$

*W_b_*: amount of APT in oil; *W_a_*: total amount of APT.

#### 2.6.2. Morphology of APT-IE

The transmission electron microscopy (TEM) (JEM-2100, JEOL Co. Ltd, Japan) was used for the observation of morphology and particle size of APT-IE. The sample was diluted 1:50 (v/v) with ultrapure water before being added onto a copper grid. Then the thin liquid film was negatively stained by adding a drop of 2% phosphotungstic acid solution. The sample was placed at room temperature until dried before being TEM observed.

### 2.7. Drug release in vitro

Dialysis method was used for the drug release *in vitro*. The PBS containing 0.5% Tween 80 (pH = 7.4) was used as release medium. APT-IE (1 mL) and APT-SL (1 mL) were taken into dialysis bag respectively, with the water bath at 37 °C. Samples were taken at 0.5, 1, 2, 4, 6, 8, 10, 12 and 24 h, each time 2 mL of release medium was removed and added with 2 mL of fresh release medium. The concentration of APT was determined by HPLC and then calculated the cumulative release rate.

### 2.8. Hemolysis experiment

The test was performed to measure the hemolytic potential to assess the safety of APT-IE. The red blood cells from rabbits were diluted with normal saline and configured as a 2% red blood cell suspension. The design of hemolysis tests of APT-IE and APT-SL were shown in Table 2. The number 6 and 7 were used as negative control and positive control respectively. These samples were incubated in a constant-temperature water bath at 37 °C for 4 h, then observe hemolysis and evaluate the safety of APT-IE and APT-SL (25% propylene glycol solution containing 0.5% polysorbate 80).

**Table 2.** Hemolysis test number design.

No	1	2	3	4	5	6	7
2% erythrocyte standard dispersion (mL)	2.5	2.5	2.5	2.5	2.5	2.5	2.5
0.9% saline injection solution (mL)	2.0	2.1	2.2	2.3	2.4	2.5	0
Distilled water (mL)	0	0	0	0	0	0	2.5
APT-IE/APT-SL (mL)	0.5	0.4	0.3	0.2	0.1	0	0

### 2.9. Stability study

#### 2.9.1. Clinical compatibility

APT-IE is a high-concentration emulsion that needs to be thinned with 0.9% sodium chloride (NaCl) or 5% glucose (G) solution before clinical use. Aseptic withdrawal of 18 mL APT-IE with transfer into 130 mL of 0.9% NaCl or 5% G solution, then observe the appearance and determine the particle size, pH, osmotic pressure and drug concentration of APT-IE at 0, 0.5, 1, 2, 4, and 6h.

#### 2.9.2. Long term stability

The long term stability of APT-IE was investigated at 25 ± 2 °C and 4 ± 2 °C for more than 6 months. The samples were evaluated by the changes in appearance, particle size, pH, zeta potential, osmotic pressure and drug content respectively.

### 2.10. In vivo study

#### 2.10.1. Pharmacokinetic investigation of APT-IE and APT-SL

The Rats (180 ± 20 g) were divided into two groups (*n* = 6). Both groups were injected with APT-IE or reference APT-SL (11.7 mg/kg) via tail vein. The heparinized tubes were then used to collect blood samples at 0.033, 0.083, 0.25, 0.5, 1, 2, 3, 4, 6, 8, 10 and 12 h by retroorbital vein, respectively. Then the upper plasma was collected from the whole blood by centrifuging it at 12000 rpm for 10 min.

#### 2.10.2. Tissue distribution study of APT-IE and APT-SL

Seventy-two rats were randomly divided into twelve groups. Six groups were administered with APT-IE at a dose of 11.7 mg/kg via the tail vein. The other groups received the same dose of APT-SL. The rats were sacrificed at 5, 10, 60, 180, 360, and 720 min after drug administration, with the brain, heart, liver, spleen, lung and kidney removed subsequently. The tissues were washed, drained, weighed, and homogenized with 0.9% saline at three times the weight of the tissues. The homogenized solution was then separated by centrifuging it at 1500 rpm for 15 min and the supernatant was stored frozen until analysis.

### 2.11. Plasma and tissue sample analysis

#### 2.11.1. Sample disposal

Took 100 μL of plasma or homogenized solution, 400 μL methanol and 20 μL butylparaben (internal standard) were added. The mixture was vortexed for 10 min, then centrifuged at 14000 rpm for 12 minutes to collect the supernatant. Subsequently, the sample was dried under the protection of nitrogen and reconstituted with methanol. The concentration of APT in plasma or homogenized solution was determined by HPLC equipped with an Agilent C_18_ column (150 mm ×4.6 mm, 5 μm particle size) at 40 °C. The mobile phase was methanol-water (67:33, v/v) at a flow rate of 1.0 mL/min. The wavelength of the UV detector was set at 220 nm.

#### 2.11.2. Pharmacokinetic parameters

The data were analyzed by Winnonlin version 6.4 software (Pharsight Corporation, San Diego, USA) based on a non-compartment model. Moreover, the Winnonlin software was also used to calculate the *AUC*_(0–∞)_ concentration of APT in each organ.

The targeting of each organ was compared based on the following parameters: Relative uptake rate (*R_e_*), Targeting efficiency (*T_e_*%), Relative targeting efficiency (*R_Te_*) and Peak concentration ratio (*C_e_*). The parameters were calculated as follows:

(5) $$R_{e}=\frac{{\left(AUC_{t}\right)}_{l}}{{\left(AUC_{t}\right)}_{s}}$$

(6) $$T_{e}\%=\frac{AUC_{t}}{\sum AUC_{t\prime }}*100\%$$

*AUC_t_* refers to *AUC* of the tissue. *AUC_t’_* refers to the total number of tissues, including target tissue.

(7) $$R_{Te}=\frac{{\left(T_{e}\right)}_{l}}{{\left(T_{e}\right)}_{s}}$$

(8) $$C_{e}=\frac{{\left(C_{\max}\right)}_{l}}{{\left(C_{\max}\right)}_{s}}$$

Each corner marker has the following meanings: *t* refers to the tissue, *l* and s refer to the lipid emulsion and solution, respectively.

#### 2.11.3. Statistical analysis

T-tests were used to estimate the significant difference between pharmacokinetic parameters of the APT-IE and APT-SL, and *P* < 0.05 indicated that the difference was statistically significant.

## 3 Results and discussion

### 3.1. Analysis of the physical and chemical properties of APT

**Figure 1.**
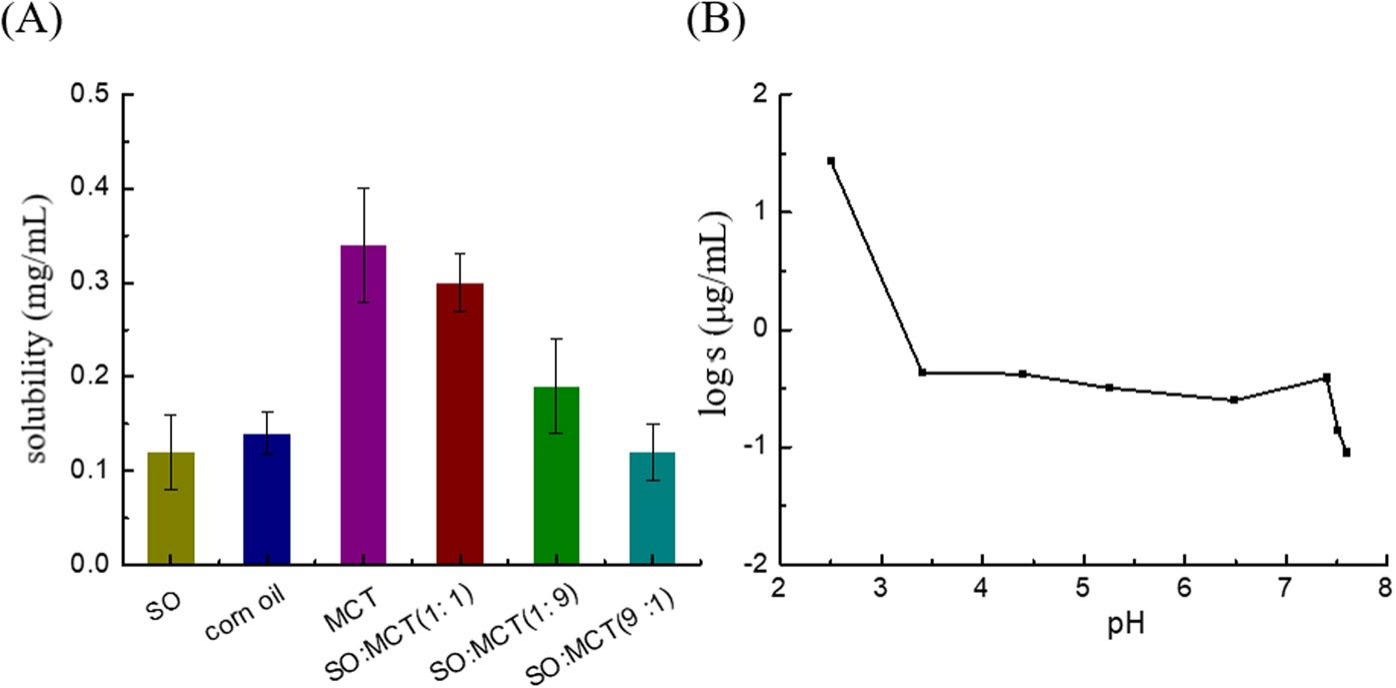
Solubility in oil (A) and apparent solubility in PBS (B) of APT (*n* = 3).

As APT with low permeability and poor solubility in intestine, the solubility of APT in water was determined of 0.62 ± 0.024 μg/mL. The solubility of APT in oil (Figure 1(A)) is approximately one hundred times higher than that of APT in water. However, the solubility in oil still failed to meet the drug loading requirements of the preparation. The relationship between the solubility of APT on a natural logarithmic scale and a series of pH values was shown in Figure 1(B). The solubility of APT in an acidic buffer solution is significantly higher than that of APT in the other solutions. APT is an alkaline compound with a *pKa* value *of* 9.7 within the pH range from 2 to 12 (Wu et al., 2004). And the weakly basic drugs tend to have a higher dissolution rate at lower pH.

Due to the extreme hydrophobicity of APT, it is necessary to control the volume of the aqueous phase to 7 times the volume of the *n*-octanol phase so that the concentration in the water is not too low to be determined. The log value of the partition coefficient is 3.37 ± 0.04 by this method. As anticipated, APT had an appropriate partition coefficient. If the log*P* value is too large (> 6) or too small (<3), the drug transport characteristics may be poor (Whateley, 2002).

### 3.2. Investigation of the APT-PC

Complexes formed by drugs and phospholipids can significantly increase the solubility and bioavailability of the poorly water-soluble drugs (Martinez & Amidon, 2002). And different ratios of drug-phospholipids have been attempted to achieve the maximum drug loading of the phospholipid complex (Yue et al., 2010). At the present study, As the ratio of APT and EL increased from 1:1 to 1:17.5, the drug loading increased significantly. The drug loading of APT:EL (1:10) was (94.44 ± 2.78)%, which can meet the drug load requirement. The mass ratio of APT to EL had a greater effect on the recombination rate, and the recombination rate increased with the increase in the EL ratio. The APT-EL ratio of 1:10 was determined considering that the smaller proportion of excipients was better without affecting the recombination rate.

#### 3.2.1. XRD analysis

**Figure 2.**
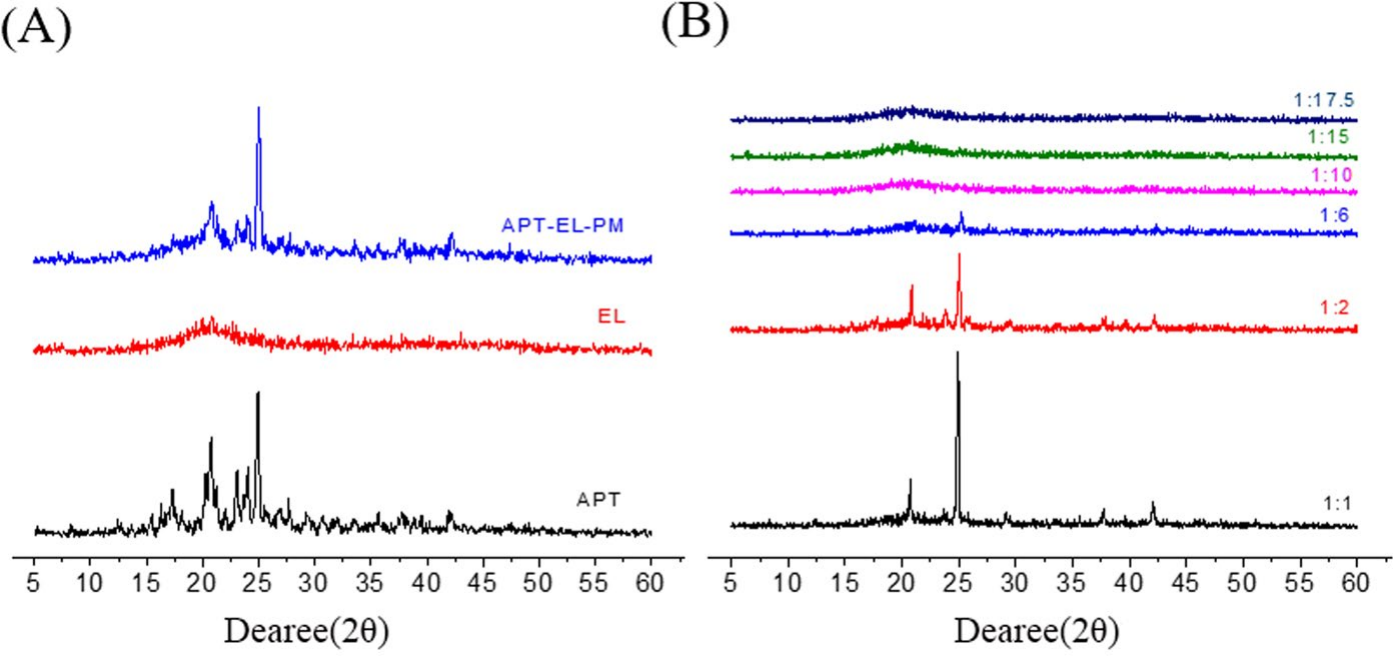
XRD spectra of APT, EL, APT-EL-PM (A) and different ratios of APT-PC (B).

In the XRD, the large diffraction peaks homologous to the crystalline drug completely disappeared or the intensity decreased after forming a phospholipid complex with the phospholipid (Gnananath et al., 2017). The XRD characteristics of APT, EL, APT-EL-PM and APT-PC are shown in Figure 2. There was a distinct crystalline peak in the diffraction pattern of APT (2θ = 24–25 °C), which meant that crystallinity existed. This series of sharp diffraction peaks were also observed in the APT-EL-PM. The XRD pattern of APT-PC with different mass ratios showed that the crystalline peak intensity of APT (2*θ* = 24–25 °C) gradually decreased. From the phospholipid complex sample (1:10), the representative diffraction peak of APT disappeared, which showed that APT was no longer in a crystalline state but in an amorphous state (Martinez & Amidon, 2002).

#### 3.2.2. FT-IR analysis

**Figure 3.**
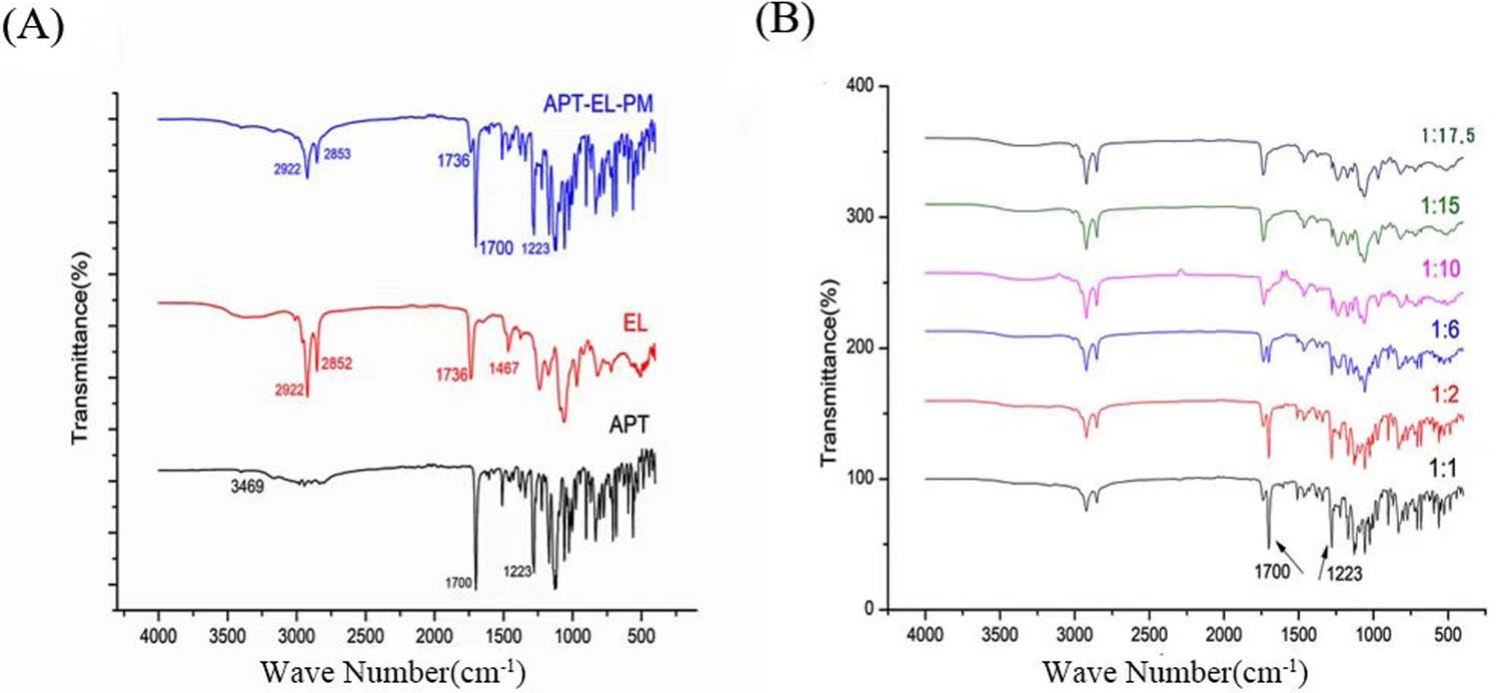
FT-IR spectra of APT, EL, APT-EL-PM (A) and different ratios of APT-PC (B).

As shown in Figure 3, the FTIR spectrum of APT had absorption bands at 1700 cm^−1^ and 3469 cm^−1^, indicating the presence of -C = O and -NH groups. The EL spectrum had absorption bands at 1736 cm^−1^ and 1467 cm^−1^, indicating the presence of -C = O and = C-H groups. The APT-EL-PM showed mainly characteristic peaks at 1700 cm^−1^, 1736 cm^−1^ and 1467 cm^−1^, which indicated the presence of APT -C = O and EL -C = O, =CH, manifesting that APT was present as a single substance instead of a complex with phospholipids. From the FTIR spectrum of APT-PC with different mass ratios, the FTIR spectrum of -C = O (1700 cm^−1^) of APT almost disappeared when the mass ratio was 1:10. We speculated that the phospholipid complex was formed by the interaction of the site with high electronegativity (-C = O) in APT and the polar end in the phospholipid molecule. Furthermore, there was no new absorption peak in the APT-PC spectrum, indicating that the phospholipid complex did not form new compounds.

#### 3.2.3. DSC analysis

**Figure 4.**
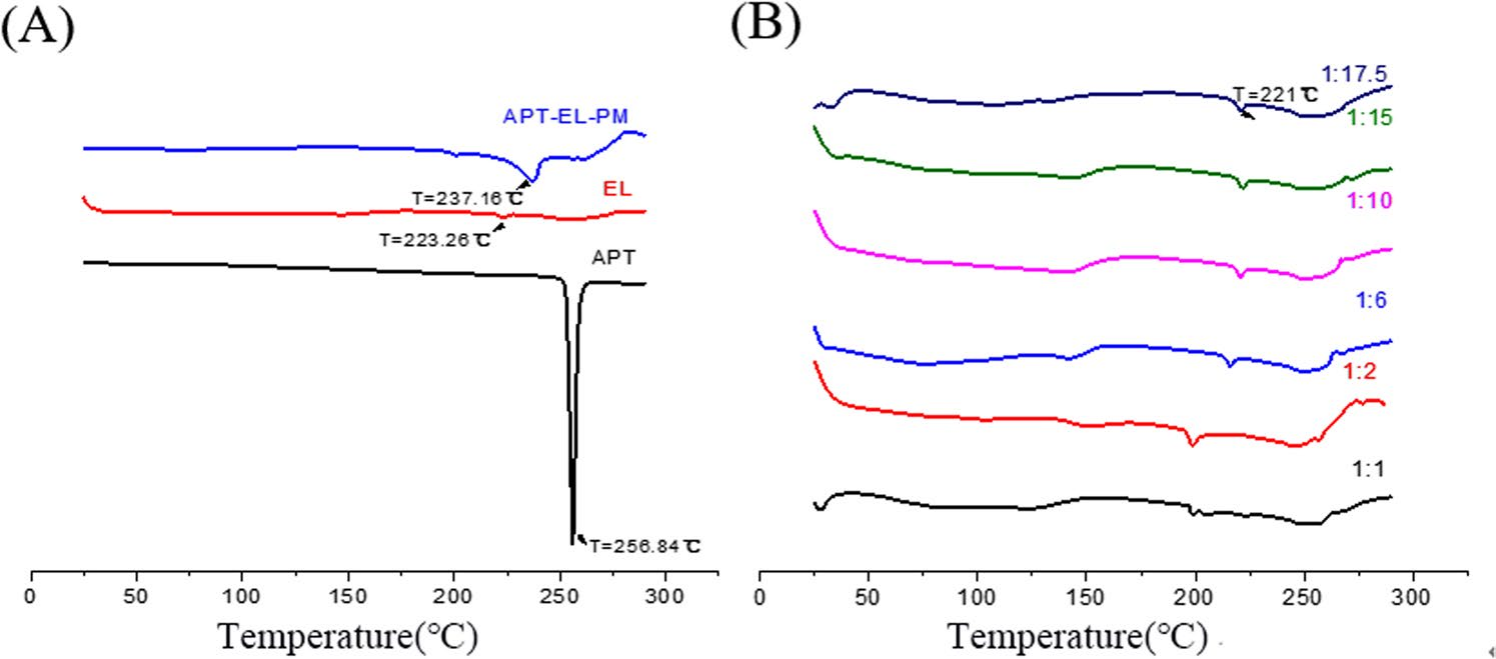
DSC thermograms of APT, EL, APT-EL-PM (A) and different ratios of APT-PC (B).

The maximum information about compatibility of drugs and excipients can be provided by DSC analysis (Lu et al., 2009). The melting peak of the compound disappeared, with a phase transition temperature of the phospholipid decreased in the phospholipid complex, which indicating the formation of the amorphous state of the drug (Kuche et al., 2019). The DSC characteristics of APT, EL, APT-EL-PM and APT-PC with different mass ratios are presented in Figure 4. APT showed sharp endothermic peaks at 256.84 °C, which was the melting point of APT. The endothermic behavior of EL near 223.26 °C may be due to the occurrence of phase transformation. In the DSC curve of APT-EL-PM, there were small sharp absorption peaks at 237.16 °C, which were lower than the melting temperature of APT in the APT curve. Moreover, the EL phase transition temperature occurs at a lower temperature than the sharp peak at 223.26 °C in the phospholipid curve because when the temperature rises, the interaction of APT and the EL moiety forms a phospholipid complex that lowers the EL phase transition temperature (Lu et al., 2009). We found the DSC results were consistent with the XRD and FTIR results, which confirmed that the drug phospholipid complex (APT-PC) was formed between APT and phospholipids.

Therefore, the hydrogen bonds and intermolecular forces was possible contributed to the formation of APT-PC. With the ratio of drug to EL increased to 1:10, the APT was mostly encapsulated by EL. Due to the structure of APT-PC could effectively improves the solubility and stability of APT, APT-PC with a ratio of APT to EL of 1:10 could be used to prepare emulsions.

### 3.3. Optimizing formulation

#### 3.3.1. Choices of oil and the percentage of EL

MCT not only can decrease the viscosity of the oil phase (Jumaa & Müller, 2001) but also improve the solubility of the drug in LCTs. However, the APT-IE prepared by MCT in this study had the smallest particle size and the largest viscosity, while emulsion prepared by LCT had the largest particle size and the smallest viscosity. Moreover, the viscosity of APT-IE prepared by the LCT and MCT mixture oil phase was also higher than that LCT alone. Finally, soybean oil was chosen as the oil phase in this experiment. In addition, the amount of EL had a great effect on the *EE* (%) of APT-IE. When the amount of EL is 7.2%, the *EE* (%) of APT-IE can meet the requirements for clinical application with the particle size approximately 100 nm. This is because EL not only can increase the solubility of APT, but also as a emulsifier can reduce the interfacial free energy and form a strong emulsifying membrane to make the emulsion more stable. Therefore, 7.2% EL was added as an emulsifier to ensure sufficient drug loading and stability of APT-IE for further investigation.

#### 3.3.2. Response surface optimization of APT-IE

The percentages of sodium oleate, poloxamer 188 and soybean oil were investigated by CCD-RSM. To select a suitable RSM, analysis of variance (ANOVA) was performed on the response value. The *P*-values of the model and various factors were both less than 0.0001, indicating that the quadratic polynomial regression models were prominent and valid. In this model, the correlation coefficient *R*^2^, *F*-value and lack of fit for the *P*-value were 0.9833, 4.00 and 0.0772, respectively, which confirmed the goodness-of-fit and applicability of the model.

The equation for calculating the average particle size is as follows:

(9) Y=94.39+21.22X1−45.40X2+3.11X3+52.25X1X2+2.53X1X3+5.76X2X3−86.38X12−57.47X22−0.36X32

**Figure 5.**
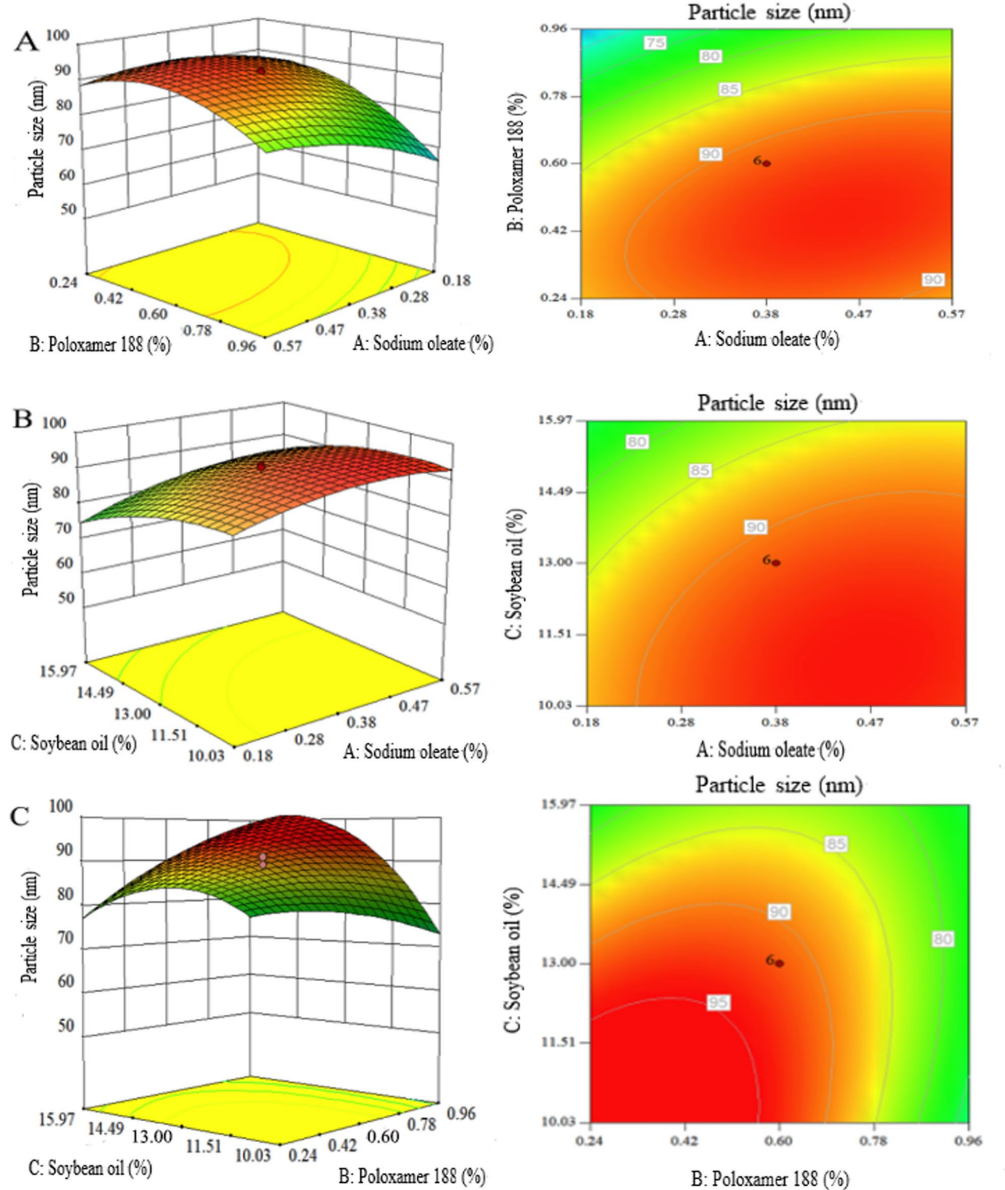
The response surface plots show the effects of sodium oleate, oil phase content and Poloxamer 188 content on the particle size of APT-IE.

Figure 5 shows the three-dimensional (3D) response surface and two-dimensional (2D) contour maps obtained from the response surface regression equation, the most direct and simple way to represent the effect of arguments on particle size (Ni et al., 2015). In this case, the interactions of X_1_X_2_ and X_2_X_3_ were significant.

The formulation with small particle size tends to be more viscous and could cause pain during intravenous injection (Jumaa & Müller, 1998). Taking into account the viscosity and stability of emulsion, the optimum formulation predicted was as follows: 0.56% sodium oleate, 10.02% soybean oil and 0.24% poloxamer 188. Compared the particle size between model prediction values and the measured value of optimized formulation, the percent deviations of 3.43% indicating that the optimized formulation was reliable and reasonable.

### 3.4. Preparation investigation

**Figure 6.**
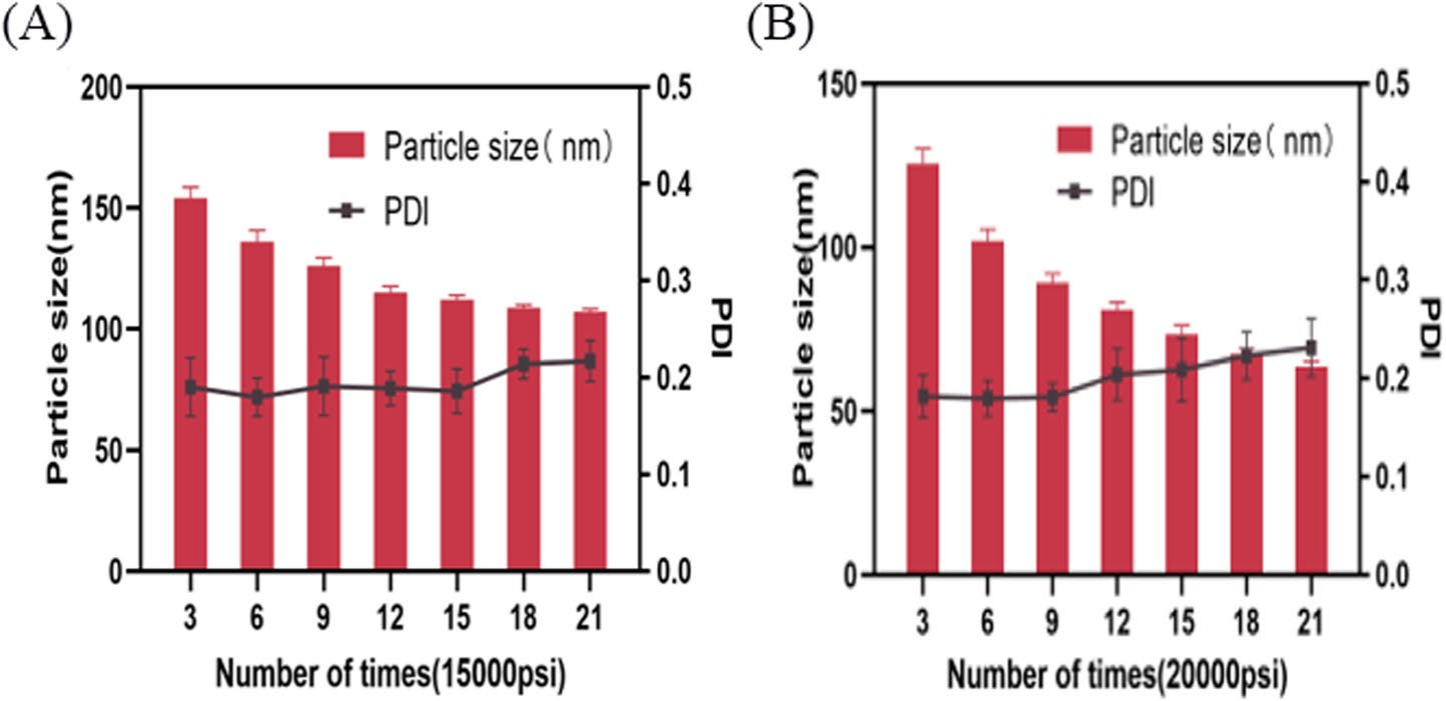
Effect of homogenization pressure and cycles on the particle size and PDI of APT-IE (*n* = 3).

In this experiment, two pressure levels (15000 psi and 20000 psi) and 7 high-pressure homogenization cycle levels were investigated. As shown in Figure 6, The homogenization process can reduce the particle size and make the particle size distribution more uniform (Jiao et al., 2002). However, the particle size remained constant when the system tended to be uniformly balanced, indicating that the maximum dispersibility and minimum particle size were achieved at the given homogenization pressure (Keck & Müller, 2006). Furthermore, the increase in PDI with 15000 psi (15 cycles) and 20000 psi (9 cycles) may be due to overpressure or prolonged homogenization leading to destroy the uniformity of the emulsion (Yu et al., 2008). A particle sizes of 89.14 nm was obtained at 20000 psi for 9 cycles, and 112.1 nm was obtained at 15000 psi for 15 cycles. Due to smaller emulsion particle size can maintain good stability, thus we decided to set the pressure at 20000 psi for 9 cycles in this study.

The sterilization method had a significant effect on the characterization and appearance of the APT-IE system. After moist heat autoclaving, the physical properties and appearance of the samples were obviously changed: the color of APT-IE became dark, the liquidity decreased, and some demulsification occurred. Additionally, the drug content and pH value of APT-IE decreased significantly. This result happened may due to the decomposition of phospholipids at high temperature and an increase of free fatty acids. However, no significant changes in appearance, particle size, drug content, and pH value were found after APT-IE sterilized by a 0.21 μm Nalgene® nylon filter. Therefore, the poor compatibility between APT cannot tolerate humid heat autoclaving (Zhang et al., 2020), but it can be sterilized by a 0.21 μm Nalgene® nylon filter.

### 3.5. Characterization of APT-IE

**Figure 7.**
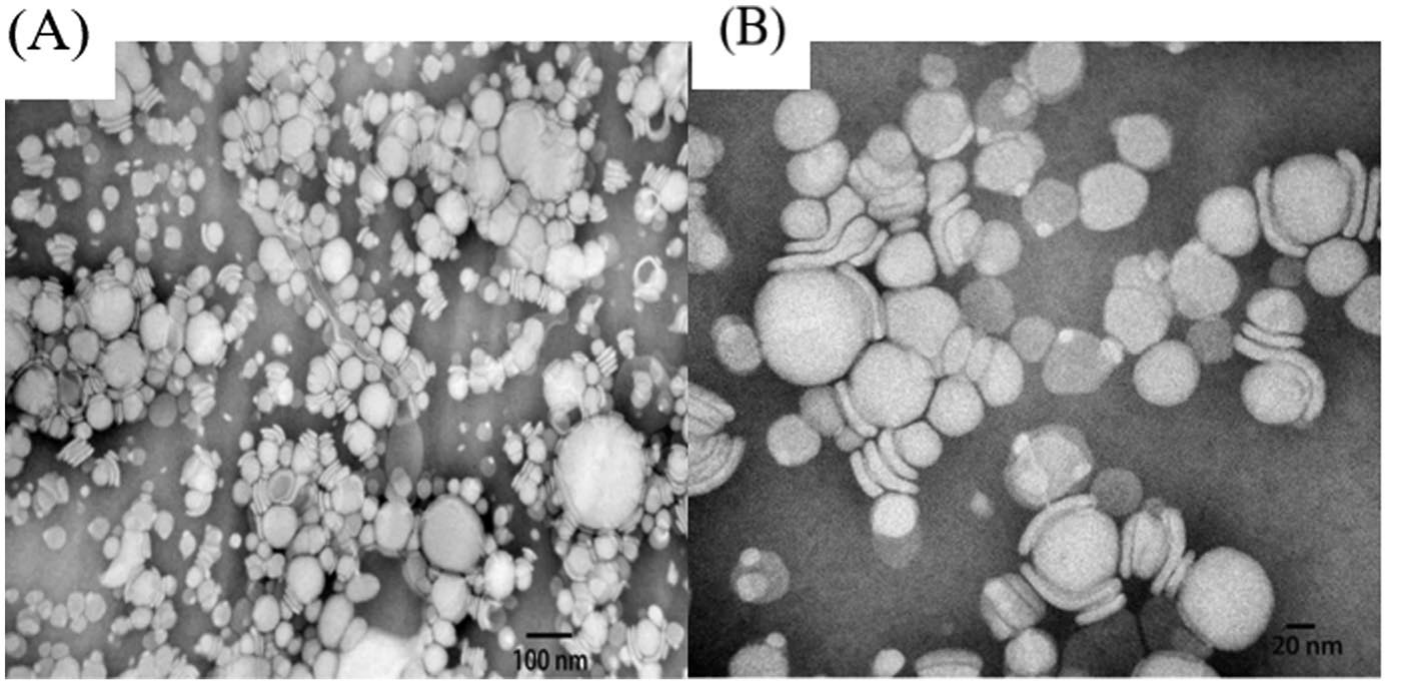
TEM image of APT-IE (A, B)

**Figure 8.**
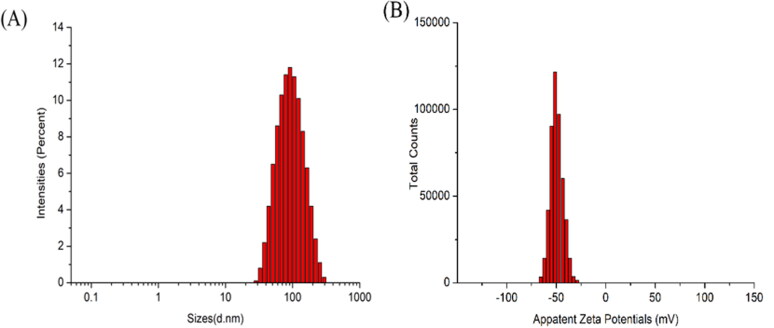
Particle size (A) and zeta potential (B) distribution of APT-IE

The DL of APT-IE was 7.08 ± 0.16 mg/mL, the particle size, PDI, zeta potential, pH value, osmotic pressure and encapsulation efficiency were found to be 82.83 ± 1.89 nm, 0.243 ± 0.008, −59.0 ± 2.54 mV, 8.21 ± 0.07, 301 ± 2.15 mOsmol/kg, and 98.84 ± 1.43% respectively. TEM image of APT-IE was presented in Figure 7(A),(B). The particle size and zeta potential distribution of APT-IE are shown in Figure 8(A),(B). The appearance of APT-IE is close to a sphere or ellipsoid morphology, TEM measurements were almost consistent with the results obtained above. APT-IE prepared in this paper has similar physical and chemical properties to commercial preparations (CINVANTI^®^).

### 3.6. Drug release

**Figure 9.**
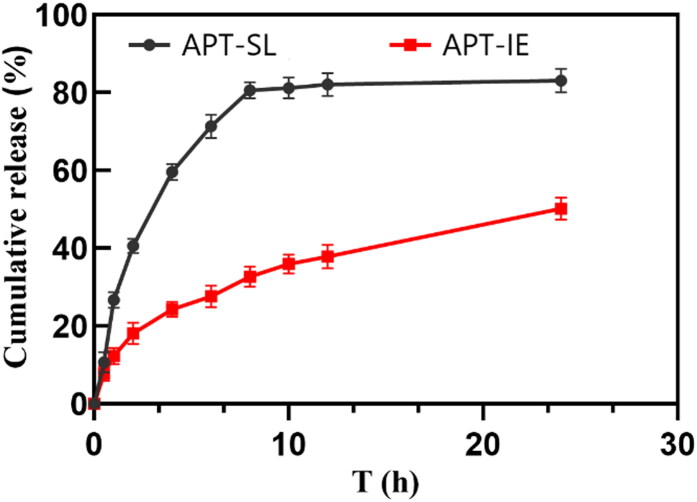
The cumulative release profiles of APT-IE and APT-SL.

The release profile of APT-IE and APT-SL was shown in Figure 9. During the initial 8 h, the cumulative release percentage of APT from APT-IE and APT-SL was 32% and 80%, respectively. After 24h, the cumulative release of APT from APT-IE and APT-SL was 50% and 83%, respectively. Thus it could be predicted that APT-IE had a certain slow-release effect. This results may due to the release of APT from the oil phase was a slow process that including breaking emulsion and diffusion. The release result of APT from APT-IE was fitted with different release kinetic equations. The *R^2^* of Zero order release, first release and Higuchi equations were 0.8410, 0.9121 and 0.9902, respectively. Therefore, the release kinetic of APT-IE was followed the Higuchi equations.

### 3.7. In vitro safety evaluation

**Figure 10.**
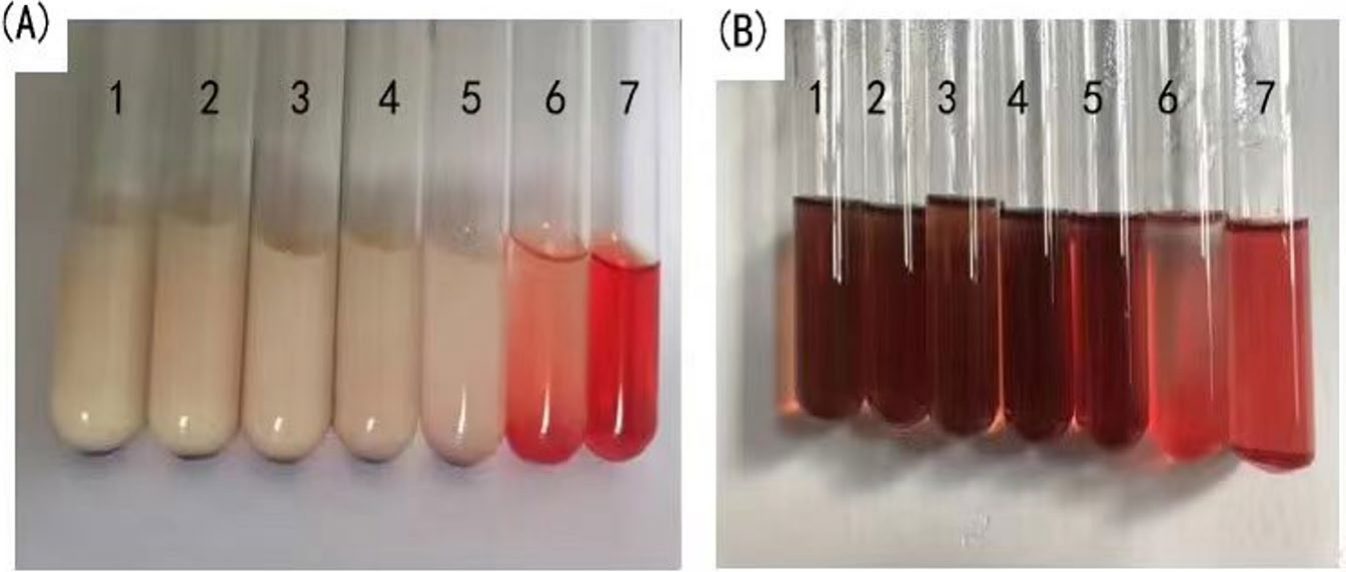
Status of hemolysis safety evaluation with APT-IE (A) and APT-SL (B)

An *in vitro* hemolysis test is considered as a reliable and direct method to predict the safety of injectable preparations. Complete hemolysis was observed in number 7 positive control group, while in number 6 negative control group, the test tube was nearly transparent with the red blood cells sedimentation (Figure 10). The other five tubes of APT-IE were consistent with the negative control tube, indicating that APT-EL did not induce hemolysis. However, there was a deep red color in the five tubes of APT-SL group and no erythrocyte were observed in the bottom. It may be attributed to the addition of organic reagent in the APT-SL. This result demonstrated that APT-IE was safer than APT-SL.

### 3.8. Stability experiments

#### 3.8.1. Compatibility stability

The compatibility stability of APT-IE in combination with 5% G and 0.9% NaCl was investigated within 6 hours. The samples had no color change, precipitation or gas evolution during the 6-hour test period, and there also had no obvious change in drug concentration, pH value or osmotic pressure in both solutions. In addition, the particle size of the samples did not change in 5% G, while slightly changed (0 h: 81.81 ± 1.46 nm, 6 h: 92.65 ± 1.24 nm, *P < 0.05, significant) in 0.9% NaCl. This result occurred may be related to the presence of cations (Na^+^, H^+^), the negatively charged droplets adsorb the positive charges and the repulsive force between the droplets is decreased. Therefore, 5% G was suggested for the application in the clinical compatibility of APT-IE.

#### 3.8.2. Long-term stability investigation

Lipid emulsion systems are inherently unstable, various unstable processes often occur during storage (Chu et al., 2012). Therefore, the investigation of long-term stability of APT-IE is necessary, which can guarantee the effectiveness and safety of the formulation. The long-term stability of APT-IE was carried out at 25 ± 2 °C and 4 ± 2 °C for 6 months, and the results were shown in Table 3. No significant changes of each parameter were found at 4 ± 2 °C for 6 months. However, the pH value and content of the samples decreased significantly after storage at 25 ± 2 °C for 6 months, this result happened possibly due to the oxidation and hydrolysis of phospholipids caused the leak of APT (Mozuraityte et al., 2006). Moreover, soybean oil also could be slowly oxidized to produce free fatty acids. Hence, the APT-IE can storage stable at 4 ± 2 °C for at least 6 months and the long-term stability study is still in progress.

**Table 3.** Characterization of optimum APT-IE during 6 months of investigation at 25 ± 2 °C and 4 ± 2 °C (*n* = 3).

Characterization of APT-IE	0 time	Storage at 25 ± 2 °Cfor 6 months	Storage at 4 ± 2 °Cfor 6 months
Physical appearance	off-white to amber liquid(Good)	Dark yellow, Poor liquidity(Bad)	off-white to amber liquid(Good)
Particle size(nm)	82.83 ± 1.28	81.80 ± 1.43	82.09 ± 1.26^#^
PDI	0.243 ± 0.002	0.206 ± 0.002	0.205 ± 0.005^#^
pH	8.21 ± 0.07	7.24 ± 0.06*	8.18 ± 0.04^#^
The existence of drug crystal	NF(Not find)	NF	NF
Zeta potential(mv)	−59.00 ± 1.59	−55.04 ± 2.04	−56.50 ± 1.29^#^
Drug content(%)	98.44 ± 1.43	75.80 ± 2.15*	97.89 ± 1.75^#^
Osmotic pressure(mOsmol·kg^–^1)	301 ± 2.15	305 ± 1.80	299 ± 1.73^#^

^#^Comparison 0 time, *P* > 0.05, not significant; ^*^Comparison 0 time, *P* < 0.05, significant.

### 3.9. In vivo pharmacokinetic study

**Figure 11.**
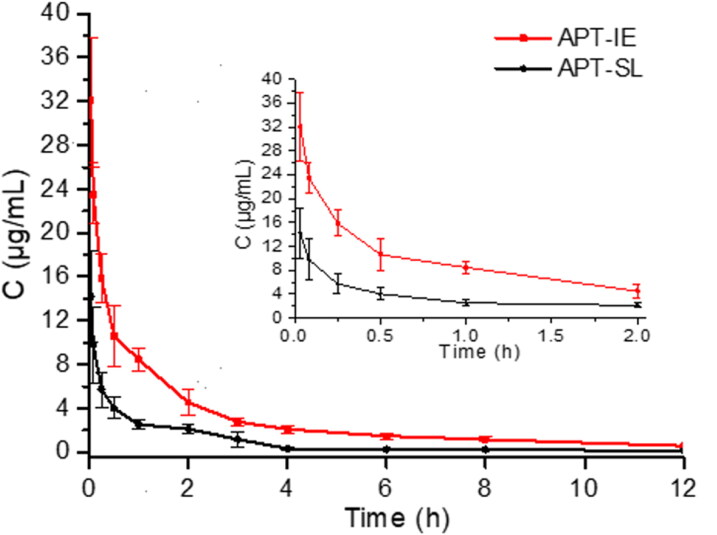
Rat plasma concentration versus time curves for APT-IE and APT-SL after intravenous administration at a dose of 11.7 mg/kg.

After intravenous injection of APT-IE and APT-SL, there were no obvious side effects in the two groups. The mean plasma concentration-time profiles of APT-IE and APT-SL are shown in Figure 11, the pharmacokinetic parameters are presented in Table 4. The *AUC_0–∞_* and the *C*_max_ of the APT-IE group were both approximately 3-fold higher than that of the APT-SL. This result may due to the APT is slowly released from the oil phase, the *CL* value of APT-IE was also lower than that of APT-SL, which implied that APT-IE can prolong the residence time of APT in blood. Therefore, we can conclude that the bioavailability of APT could be significantly improved after preparing into lipid emulsions.

**Table 4.** Pharmacokinetic parameters after intravenous administration of APT-IE and APT-SL to rats (*n* = 6).

Parameter	APT-IE	APT-SL
C_max_ (μg·mL^−1^)	32.95 ± 5.09*	14.2 ± 4.12
AUC_(0–∞)_ (h·μg·mL^−1^)	39.72 ± 3.17*	12.72 ± 2.42
MRT_(0–t)_ (h)	2.72 ± 0.09*	2.05 ± 0.22
T_1/2_ (h)	4.14 ± 1.08	4.88 ± 1.87
Vd (L·kg^−1^)	1.76 ± 0.42*	6.49 ± 2.48
CL (L·h·kg^−1^)	0.30 ± 0.02*	0.94 ± 0.14

* Compared APT-SL, *P* < 0.05, significant.

### 3.10. Tissue distribution study

**Figure 12.**
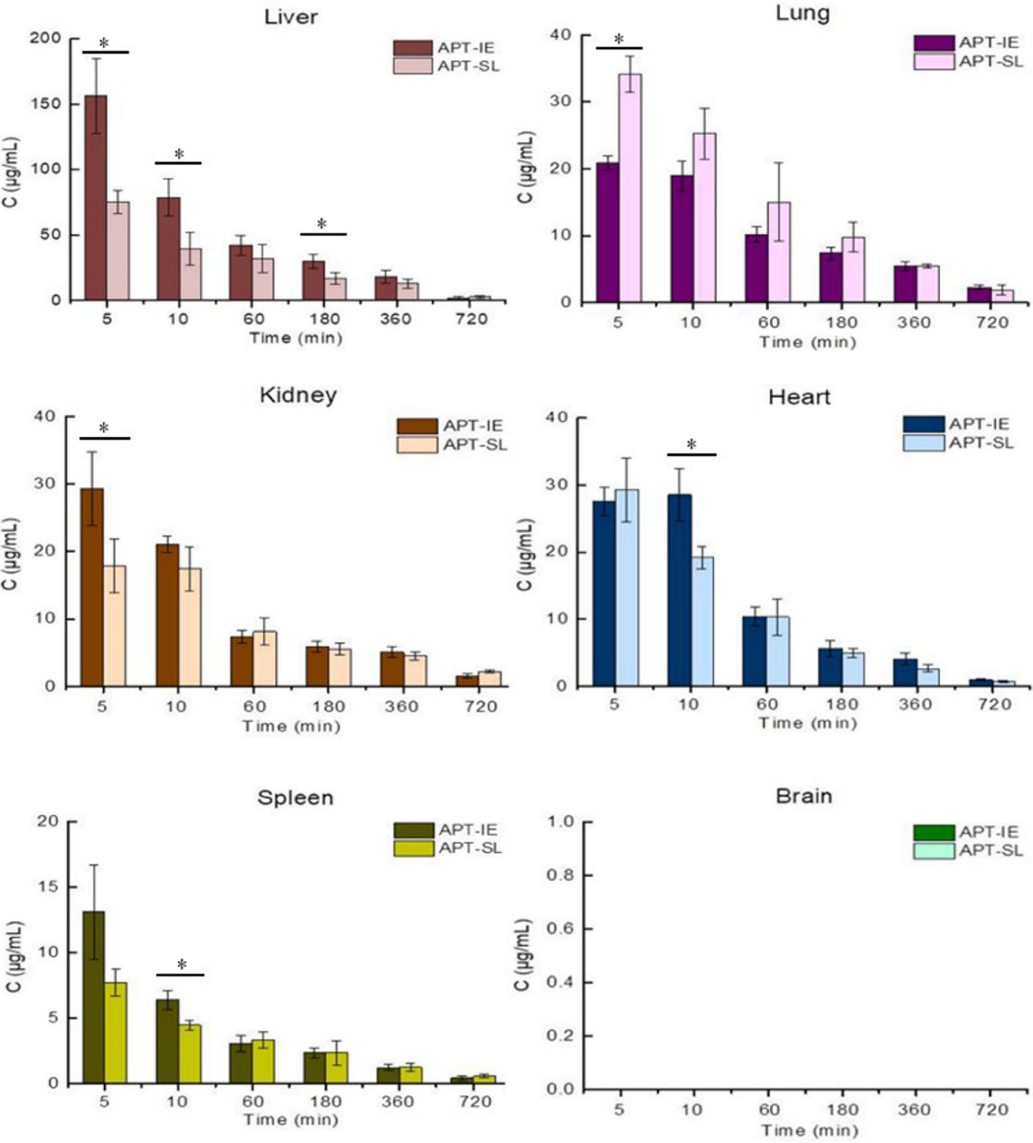
Tissue distribution of APT after intravenous administration of APT-IE and APT-SL in rats (*P < 0.05, *n* = 6).

Figure 12 shows the mean concentration-time profiles of APT in six tissues after intravenous administrated with APT-IE and APT-SL, and the *AUC*_0−∞_ in various tissues were shown in Table 5, respectively. APT could enter the brain through the blood-brain barrier, however, in the present research, no drug was detected in the brain, which might attribute to the hypothesis NK-1 receptor might exist in the cerebrospinal fluid and lost in the process of brain collection, so APT could not be detected in the brain tissue (Yanagikawa, 1982). The order of concentrations for each tissue after intravenous administration of APT-IE and APT-SL at five minutes was as follows: liver > kidney≈heart > lung > spleen, and liver > lung≈heart > kidney > spleen, respectively. After intravenous administration of APT-IE, the *AUC*_0–∞_ for APT-IE was 1.46 and 1.19-fold in the liver and heart, respectively, compared to ATP-SL. When emulsions are injected intravenously, they are more easily absorbed by the reticuloendothelial system such as the liver (Mizushima et al., 1982). The concentration and AUC of APT in the liver were the highest at different times, indicating the highest uptake rate of liver and the possibility of liver targeting of the drug. Table 6 lists the four targeting parameters: R_e_, T_e_ and R_Te_ were calculated by the AUC_0–∞_ of APT-IE and APT-SL; C_e_ was calculated by the maximum concentration (*C_max_*) of APT in the same tissue after administration of APT-EL and APT-SL. R_e_*>*1 manifests the emulsion possess targetability, and R_e_*≤*1 shows no targetability, In addition, the larger R_e_ value indicates the better targetability of APT-IE (Luo et al., 2016). R_Te_ represents the multiple of the R_e_ for APT-IE compared with APT-SL. A larger value of C_e_ implies a greater change in biodistribution after administration of APT-IE. In this study, APT-IE possesses favorable liver-targeting efficiency with R_e_: 1.52; T_e_(%): 55.71%, which are significantly higher than the R_e_ and T_e_(%) of other organs. The R_Te_ value of liver and heart were larger than 1.00, which indicates enhanced targeting of both tissue by APT-IE compared to APT-SL. At present, APT is widely used as an NK-1 receptor antagonist for the treatment of CINV (Navari, 2004). However, it has been reported that APT induces an antitumor influence by leading to apoptosis in tumor cells (Muñoz & Coveñas, 2020). The obvious liver targeting of APT-IE may create good prospects for its antitumor effect in the future.

**Table 5.** Comparative values of AUC_(0–∞)_ in various tissues after intravenous administration of APT-IE and APT-SL to rats (*n* = 6).

Tissues	Liver	Lung	Kidney	Heart	Spleen
APT-IE (h·μg·mL^−1^)	291.13 ± 17.19*	91.32 ± 7.74	75.37 ± 5.55	68.30 ± 5.65	24.19 ± 2.03
APT-SL (h·μg·mL^−1^)	199.02 ± 13.00	104.43 ± 5.16	84.77 ± 3.56	57.18 ± 5.01	25.23 ± 4.93

*compared APT-SL, *P* < 0.05, significant.

**Table 6.** Targeting parameters of APT-IE and APT-SL in various tissues of rats.

Parameters	Heart	Liver	Spleen	Lung	Kidney
R_e_(APT-IE)	1.18	1.52	1.02	0.81	1.03
T_e_(APT-IE)(%)	12.51	55.71	4.28	14.63	12.80
T_e_(APT-SL)(%)	11.93	40.93	4.71	20.37	13.86
R_Te_	1.05	1.36	0.90	0.71	0.92
C_e_	1.02	2.07	1.69	0.61	1.52

## 4. Conclusions

In this research, the APT-IE was prepared by optimal formulation, with the component of APT (7.2%, w/v), soybean oil (10.02%, w/v), egg yolk lecithin (14.4%, w/v), poloxamer 188 (0.24%, w/v), sodium oleate (0.56%, w/v), and sucrose (5.56%, w/v). The emulsion performed good physicochemical properties. Hemolysis tests showed that intravenous injection for APT-IE was safe and suitable. This research confirmed that APT-IE could be stably stored for at least 6 months at 4 ± 2 °C. The results of pharmacokinetic and tissue distribution studies demonstrated that APT-IE expressed increased bioavailability, and obvious liver targeting compared with APT-SL. Consequently, this research suggests that APT-IE loaded with APT-PC has broad prospects for clinical application and provide a data reference for the further study of APT intravenous emulsions.

## Supplementary Material

Supplemental Material

## Abbreviations

APT: aprepitant
APT-IE: aprepitant lipid emulsion
APT-PC: aprepitant phospholipid complex
APT-SL: aprepitant solution
APT-EL-PM: aprepitant and phospholipid physical mixture
AUC: area under curve
CCD: center composite design
CHS: cholesterol hemisuccinate
CINV: chemotherapy-induced nausea and vomiting
DSC: differential scanning calorimeter
EL: egg yolk lecithin
EE: encapsulation efficiency
FT-IR: Fourier transform Infrared
G: glucose
HPLC: high performance liquid chromatograph
IE: injectable lipid emulsion
DL: drug loading
MCT: medium-chain triglyceride
NK-1: neurokinin-1
PBS: phosphate buffered solution
PC: phospholipid complex
PDI: polydispersity index
PM: physical mixture
RA: receptor Antagonist
RSM: response surface methodology
R_e_: relative uptake rate
R_Te_: relative targeting efficiency
Rer: recombination rate
SO: soybean oil
T_e_: targeting efficiency
XRD: X-ray diffraction

## References

[CIT0001] Aapro M, Carides A, Rapoport BL, et al. (2015). Aprepitant and fosaprepitant: a 10-year review of efficacy and safety. Oncologist 20:1–13. 25795636 10.1634/theoncologist.2014-0229 PMC4391760

[CIT0002] Augustyn B, Stepien P, Poojari C, et al. (2019). Cholesteryl hemisuccinate is not a good replacement for cholesterol in lipid nanodiscs. J Phys Chem B 123:9839–45. 31674185 10.1021/acs.jpcb.9b07853

[CIT0003] Ballatori E, Roila F. (2003). Impact of nausea and vomiting on quality of life in cancer patients during chemotherapy. Health Qual Life Outcomes 1:46. 14521717 10.1186/1477-7525-1-46 PMC212194

[CIT0004] Carpentier YA, Dupont IE. (2000). Advances in intravenous lipid emulsions. World J Surg 24:1493–7. 11193713 10.1007/s002680010267

[CIT0005] Chu T, Zhang Q, Li H, et al. (2012). Development of intravenous lipid emulsion of tanshinone IIA and evaluation of its anti-hepatoma activity in vitro. Int J Pharm 42:76–88. 10.1016/j.ijpharm.2011.12.049 22226873

[CIT0006] Driscoll DF. (2006). Lipid injectable emulsions: pharmacopeial and safety issues. Pharm Res 23:1959–69. 16951994 10.1007/s11095-006-9092-4

[CIT0007] Gnananath K, Nataraj KS, Rao BG. (2017). Phospholipid complex technique for superior bioavailability of phytoconstituents. Adv Pharm Bull 7:35–42. 28507935 10.15171/apb.2017.005 PMC5426732

[CIT0008] Hörter D, Dressman JB. (2001). Influence of physicochemical properties on dissolution of drugs in the gastrointestinal tract. Adv Drug Deli Rev 46:75–87. 10.1016/s0169-409x(00)00130-7 11259834

[CIT0009] Jiao J, Rhodes DG, Burgess DJ. (2002). Multiple emulsion stability: pressure balance and interfacial film strength. J Colloid Interface Sci 250:444–50. 16290683 10.1006/jcis.2002.8365

[CIT0010] Jumaa M, Müller BW. (1998). The effect of oil components and homogenization conditions on the physicochemical properties and stability of parenteral fat emulsions. Int J Pharm 16:81–9.

[CIT0011] Jumaa M, Müller BW. (2001). Development of a novel parenteral formulation for tetrazepam using a lipid emulsion. Drug Dev Ind Pharm 27:1115–21. 11794814 10.1081/ddc-100108374

[CIT0012] Keck CM, Müller RH. (2006). Drug nanocrystals of poorly soluble drugs produced by high pressure homogenisation. Eur J Pharm Biopharm 62:3–16. 16129588 10.1016/j.ejpb.2005.05.009

[CIT0013] Kuche K, Bhargavi N, Dora CP, et al. (2019). Drug-phospholipid complex-a go through strategy for enhanced oral bioavailability. AAPS PharmSciTech 20:43. 30610392 10.1208/s12249-018-1252-4

[CIT0014] Li Y, Jin W, Yan H, et al. (2013). Development of intravenous lipid emulsion of vinorelbine based on drug-phospholipid complex technique. Int J Pharm 454:472–7. 23806812 10.1016/j.ijpharm.2013.06.032

[CIT0015] Lu Y, Zhang Y, Yang Z, et al. (2009). Formulation of an intravenous emulsion loaded with a clarithromycin-phospholipid complex and its pharmacokinetics in rats. Int J Pharm 366:160–9. 18835427 10.1016/j.ijpharm.2008.09.008

[CIT0016] Luo LH, Zheng PJ, Nie H, et al. (2016). Pharmacokinetics and tissue distribution of docetaxel liposome mediated by a novel galactosylated cholesterol derivatives synthesized by lipase-catalyzed esterification in non-aqueous phase. Drug Deliv 23:1282–90. 25417833 10.3109/10717544.2014.980525

[CIT0017] Martinez MN, Amidon GL. (2002). A mechanistic approach to understanding the factors affecting drug absorption: a review of fundamentals. J Clin Pharmacol 42:620–43. 12043951 10.1177/00970002042006005

[CIT0018] Mizushima Y, Hamano T, Yokoyama K. (1982). Tissue distribution and anti-inflammatory activity of corticosteroids incorporated in lipid emulsion. Ann Rheum Dis 41:263–7. 6896429 10.1136/ard.41.3.263 PMC1000924

[CIT0019] Mozuraityte R, Rustad T, Storr I. (2006). Oxidation of cod phospholipids in liposomes: effects of salts, pH and zeta potential. Eur J Lipid Sci Technol 108:944–50.

[CIT0020] Muñoz M, Coveñas R. (2020). The neurokinin-1 receptor antagonist aprepitant: an intelligent bullet against cancer? Cancers 12:2682. 32962202 10.3390/cancers12092682 PMC7564414

[CIT0021] Navari RM, Mosier MC. (2019). Crossover safety study of aprepitant: 2-min injection vs 30-min infusion in cancer patients receiving emetogenic chemotherapy. Onco Targets Ther 12:3277–84. 31118678 10.2147/OTT.S201609 PMC6503333

[CIT0022] Navari RM. (2004). Aprepitant: a neurokinin-1 receptor antagonist for the treatment of chemotherapy-induced nausea and vomiting. Expert Rev Anticancer Ther 4:715–24. 15485308 10.1586/14737140.4.5.715

[CIT0023] Ni Q, Gao Q, Yu W, et al. (2015). Supercritical carbon dioxide extraction of oils from two torreya grandis varieties seeds and their physicochemical and antioxidant properties. LWT-Food Sci Technol 60:1226–34.

[CIT0024] Ottoboni T, Lauw M, Keller MR, et al. (2018). Safety of HTX-019 (intravenous aprepitant) and fosaprepitant in healthy subjects. Future Oncol 14:2849–59. 29873529 10.2217/fon-2018-0311

[CIT0025] Peng C, Liu C, Tang X. (2010). Determination of physicochemical properties and degradation kinetics of triamcinolone acetonide palmitate in vitro. Drug Dev Ind Pharm 36:1469–76. 20524902 10.3109/03639045.2010.488645

[CIT0026] Sato Y, Kondo M, Inagaki A, et al. (2014). A highly frequent and enhanced injection site reaction induced by peripheral venous injection of fosaprepitant in anthracycline-treated patients. J Cancer 5:390–7. 24799957 10.7150/jca.7706 PMC4007527

[CIT0027] Tsukiyama I, Hasegawa S, Ikeda Y, et al. (2018). Cost‐effectiveness of aprepitant in japanese patients treated with cisplatin-containing highly emetogenic chemotherapy. Cancer Sci 109:2881–8. 29999572 10.1111/cas.13736 PMC6125450

[CIT0028] Wang W, Zhang W, Jiang Y, et al. (2020). Preparation of ursolic acid-phospholipid complex by solvent-assisted grinding method to improve dissolution and oral bioavailability. Pharm Dev Technol 25:68–75. 31544585 10.1080/10837450.2019.1671864

[CIT0029] Whateley TL. (2002). Drug delivery and targeting; for pharmacists and pharmaceutical scientists. J Drug Targe 10:637.

[CIT0030] Wu Y, Loper A, Landis E, et al. (2004). The Role of biopharmaceutics in the development of a clinical nanoparticle formulation of Mk-0869: a beagle dog model predicts improved bioavailability and diminished food effect on absorption in human. Int J Pharm 285:135–146. 15488686 10.1016/j.ijpharm.2004.08.001

[CIT0031] Yanagikawa A. (1982). Application of lipid particles as a novel carrier for various drugs. J Stage 2:251–7.

[CIT0032] Yu YL, Lu Y, Tang X, et al. (2008). Formulation, preparation and evaluation of an intravenous emulsion containing brucea javanica oil and coix seed oil for anti-tumor application. Biol Pharmaceut Bullet 31:673–680. 10.1248/bpb.31.673 18379061

[CIT0033] Yue PF, Yuan HL, Li XY, et al. (2010). Process optimization, characterization and evaluation in vivo of oxymatrine-phospholipid complex. Int J Pharm 387:139–146. 20005937 10.1016/j.ijpharm.2009.12.008

[CIT0034] Zhang T, Li M, Yang R, et al. (2018). Therapeutic efficacy of lipid emulsions of docetaxel-linoleic acid conjugate in breast cancer. Int J Pharm 546:61–69. 29763687 10.1016/j.ijpharm.2018.05.032

[CIT0035] Zhang X, Wei Y, Cao ZJ, et al. (2020). Aprepitant intravenous emulsion based on ion pairing/phospholipid complex for improving physical and chemical stability during thermal sterilization. AAPS PharmSciTech 21:75. 31965388 10.1208/s12249-019-1605-7

