# Supplementary material for: Preparation and in vivo evaluation of an intravenous emulsion loaded with an aprepitant-phospholipid complex
Source: Drug Deliv. 2023 Feb 27;30(1):2183834. doi: 10.1080/10717544.2023.2183834 (PMC9979997; doi:10.1080/10717544.2023.2183834)
Supplement: Supplemental Material [file IDRD_A_2183834_SM0598.docx]

**Table 1 Drug loading of phospholipid complexes with different ratios (*n*=3)**

| Ratio | 1:1 | 1:2 | 1:6 | 1:10 | 1:15 | 1:17.5 |
| --- | --- | --- | --- | --- | --- | --- |
| Rer(%) | 15.27±3.94 | 31.94±4.17 | 70.8±5.56 | 94.44±2.78 | 91.66±4.17 | 95.83±2.78 |

**Table 2 Analysis of variance (ANOVA) for the experimental results**

| Source | Sum of Squares | Df | Mean Square | *F* Value | 1. Value   Prob>F | — |
| --- | --- | --- | --- | --- | --- | --- |
| Mode | 2615.22 | 9 | 290.58 | 65.43 | <0.0001 | Significant |
| A-*X_1_* | 218.64 | 1 | 218.64 | 49.23 | <0.0001 | — |
| B-*X_2_* | 685.05 | 1 | 685.05 | 154.25 | <0.0001 | — |
| C-*X_3_* | 359.58 | 1 | 359.58 | 80.97 | <0.0001 | — |
| AB | 103.82 | 1 | 103.82 | 23.38 | 0.0007 | — |
| AC | 16.94 | 1 | 16.94 | 3.81 | 0.0794 | — |
| BC | 298.90 | 1 | 298.90 | 67.30 | <0.0001 | — |
| A^2^ | 149.96 | 1 | 149.96 | 33.77 | 0.0002 | — |
| B^2^ | 771.06 | 1 | 771.06 | 173.62 | <0.0001 | — |
| C^2^ | 142.17 | 1 | 142.17 | 32.01 | 0.0002 | — |
| Residual | 44.41 | 10 | 4.44 | — | — | — |
| Lack of Fit | 35.53 | 5 | 7.11 | 4.00 | 0.0772 | Not Significant |
| Pure Error | 8.88 | 5 | 1.78 | — | — | — |
| Cor Tatal | 2659.64 | 19 | — | — | — | — |
